# *i*CluF: an unsupervised iterative cluster-fusion method for patient stratification using multiomics data

**DOI:** 10.1093/bioadv/vbae015

**Published:** 2024-01-30

**Authors:** Sushil K Shakyawar, Balasrinivasa R Sajja, Jai Chand Patel, Chittibabu Guda

**Affiliations:** Department of Genetics, Cell Biology and Anatomy, University of Nebraska Medical Center, Omaha, NE 68198, United States; Department of Radiology, University of Nebraska Medical Center, Omaha, NE 68198, United States; Department of Genetics, Cell Biology and Anatomy, University of Nebraska Medical Center, Omaha, NE 68198, United States; Department of Genetics, Cell Biology and Anatomy, University of Nebraska Medical Center, Omaha, NE 68198, United States; Department of Genetics, Cell Biology and Anatomy, Center for Biomedical Informatics Research and Innovation, University of Nebraska Medical Center, Omaha, NE 68198-5805, United States

## Abstract

**Motivation:**

Patient stratification is crucial for the effective treatment or management of heterogeneous diseases, including cancers. Multiomic technologies facilitate molecular characterization of human diseases; however, the complexity of data warrants the need for the development of robust data integration tools for patient stratification using machine-learning approaches.

**Results:**

*i*CluF iteratively integrates three types of multiomic data (mRNA, miRNA, and DNA methylation) using pairwise patient similarity matrices built from each omic data. The intermediate omic-specific neighborhood matrices implement iterative matrix fusion and message passing among the similarity matrices to derive a final integrated matrix representing all the omics profiles of a patient, which is used to further cluster patients into subtypes. *i*CluF outperforms other methods with significant differences in the survival profiles of 8581 patients belonging to 30 different cancers in TCGA. *i*CluF also predicted the four intrinsic subtypes of Breast Invasive Carcinomas with adjusted rand index and Fowlkes–Mallows scores of 0.72 and 0.83, respectively. The Gini importance score showed that methylation features were the primary decisive players, followed by mRNA and miRNA to identify disease subtypes. *i*CluF can be applied to stratify patients with any disease containing multiomic datasets.

**Availability and implementation:**

Source code and datasets are available at https://github.com/GudaLab/iCluF_core.

## 1 Introduction

The majority of human diseases exhibit different degrees of variability in their clinical phenotypes; therefore, they require different therapeutic strategies for different patient groups. Hence, patient stratification (also known as disease subtyping) is an important step that helps dissect the heterogeneity of each subtype, and it helps develop effective treatment protocols for each subtype. Many genetically driven diseases, such as cancer, arise from genomic alterations in different organs that lead to cellular behavioral changes. The cascade of molecular changes at the DNA, RNA, protein, and metabolic levels enables the distinction of different strata of patients. Advances in genomics technologies have resulted in a generation of various high-throughput genomics data, broadly dubbed as “multiomics data,” that concern many diseases. However, the extraction of meaningful information from these large datasets, and the establishment of its disease relevance, is very challenging and often limited by the paucity of effective data integration and interpretation tools. For instance, precise medicine-based cancer treatments primarily rely on the distinctive molecular characterization of a patient, or a cohort of patients, through disease subtyping. In the case of non-small cell lung carcinoma (NSCLC), ∼30%–40% of patients show a recurrence of tumors after curative resection (Uramoto and Tanaka 2014), suggesting that these patients essentially share some common molecular events, and thus, they need to be classified differently to develop customized treatments. Here, the unmet challenge is to accurately identify disease subtypes that have different molecular and clinical features. For most heterogeneous diseases, patient stratification using molecular subtype prediction models has caught the attention of researchers. As each omic datatype represents a different modality of gene regulation, better prediction accuracy can be achieved by integrating multiomic datasets derived from the same patient. This goal can be accomplished with the development of integrative machine-learning (ML) methods for disease subtyping.

Previous attempts to solve these issues include the application of ML and data integration approaches, which used multiomics data (such as genomic, transcriptomic, and proteomics) for subtype identification. In recent years, the rapid growth of omics technologies has led to the development of several multiomic data resources, such as TCGA (www.genome.gov), ICGC (www.dcc.icgc.org), and Genotype-Tissue Expression (gtexportal.org/home/) when characterizing human cancers. The omics cohorts of large numbers of patients have improved our understanding of the molecular basis of cancer diagnosis, prognosis, and subtyping (Gao and Church 2005, Mo *et al.* 2013, Menyhárt and Győrffy 2021, He *et al.* 2023, Li *et al.* 2023). However, the dissemination of large omic datasets also imposed challenges for data integration across different omic modalities. In this context, previous ML methods that mainly focused on the use of single omic data (mainly gene expression) for clustering include neural networks (Herrero *et al.* 2001, Luo *et al.* 2004), hierarchical clustering (Makretsov *et al.* 2004, Lin *et al.* 2015), consensus clustering (Wilkerson and Hayes 2010), and other combined approaches (Daxin *et al.* 2004, McLachlan *et al.* 2002). Later, several methods were developed which focused on the integration of multiomic datasets, along with integrative clustering approaches. These methods are believed to capture disease subtypes more holistically by considering biological phenomena at various levels. For example, intNFM (Chalise and Fridley 2017) uses multiomic datasets and identifies clusters based on non-negative matrix factorization. Similarly, LRAcluster (Wu *et al.* 2015) converts a high-dimensional multiomics feature matrix into a low-dimensional matrix using a low-rank approximation approach, followed by the identification of clusters via *K*-means. These methods assume that biological events at each omic level are linearly associated.

Other methods, such as iClusterPlus (Mo *et al.* 2013), MDI (Kirk *et al.* 2012), and iCluster (Shen *et al.* 2009) utilized statistical models for integrating multiomic datasets when identifying cancer subtypes. In the same vein, more approaches, such as CIMLR (Ramazzotti *et al.* 2018), similarity network fusion (SNF) (Wang *et al.* 2014), NEMO (Speicher and Pfeifer 2015), PINS (Nguyen *et al.* 2017), and PINSPlus (Nguyen *et al.* 2019) have been developed. Such approaches focused on separately constructing a patient–patient similarity matrix from single omic datasets, followed by integration, to identify the final clusters. CIMLR incorporates multiple Gaussian kernels to generate a patient–patient similarity matrix, followed by dimensionality reduction and cluster identification. These methods are susceptible to noise due to the application of Gaussian methods for calculating similarities between patients. Subtype-GAN (Yang *et al.* 2021) is a deep-learning method that uses the Gaussian Mixture model and consensus clustering to identify cancer subtypes. Deep-learning approaches generally need larger sample sizes, which is not the case for the majority of disease datasets. NEMO has developed a strategy for utilizing partial datasets for disease subtype identification; however, it does not handle noise in the data. PINSPlus, by using a Gaussian approach, overcame the limitation of handling noise before identifying disease subtypes. Similarly, SCFA (Tran *et al.* 2020) focuses on removing data noise during multiomic integration to identify cancer subtypes and risk scores. Limitations of these approaches include their sensitivity to noise in the data. Vahabi *et al.* comprehensively discussed the usability and limitations of the existing methods (Vahabi and Michailidis 2022). Other previous reviews have also provided insights into the working methodologies of existing methods, their limitations, and future perspectives of method development in the multiomics era when subtyping human diseases, including cancer (Das *et al.* 2020, Eicher *et al.* 2020, Zhang *et al.* 2022). Overall, the current methods suffer from various limitations, such as susceptibility to noise and a lack of sensitivity, among others, which we have attempted to address here.

In this study, we developed a novel method, Iterative Cluster Fusion (*i*CluF), which identifies disease subtypes using multi-level omics information obtained from the same patient. Our approach systematically creates patient–patient similarity measures at each omic level, and it creates neighborhood matrices by passing information between different omic levels, iteratively. The approach uniquely captures, correlates, and shares information between multiomic datasets to derive final clusters using the holistic molecular profile. Although we used cancer patient data to develop and test *i*CluF in this study, this generic method can be applied to stratify patients associated with any disease, provided the availability of multiomics datasets exists.

## 2 Methods

### 2.1 Benchmark dataset and feature selection

We used Level 3 curated miRNA, mRNA, methylation, and survival data of 30 cancer types, which are available on the TCGA website. For each cancer type, we included patients with data comprising all three omic levels, their vital status, and survival information. Adopting the strategy in Hoadley *et al.* (2018) and Yang *et al.* (2021), we selected 383 miRNAs, 3217 mRNAs, and 3139 methylation features. Simultaneously, each omic dataset was filtered by removing features with zero or missing values in more than 20% of the samples. The detailed processing steps of individual data types are provided in the Supplementary Material. The final data include 30 cancer types with different numbers of samples and features (provided in Supplementary Table S1).

### 2.2 *i*CluF: data integration and clustering

**Figure 1.**
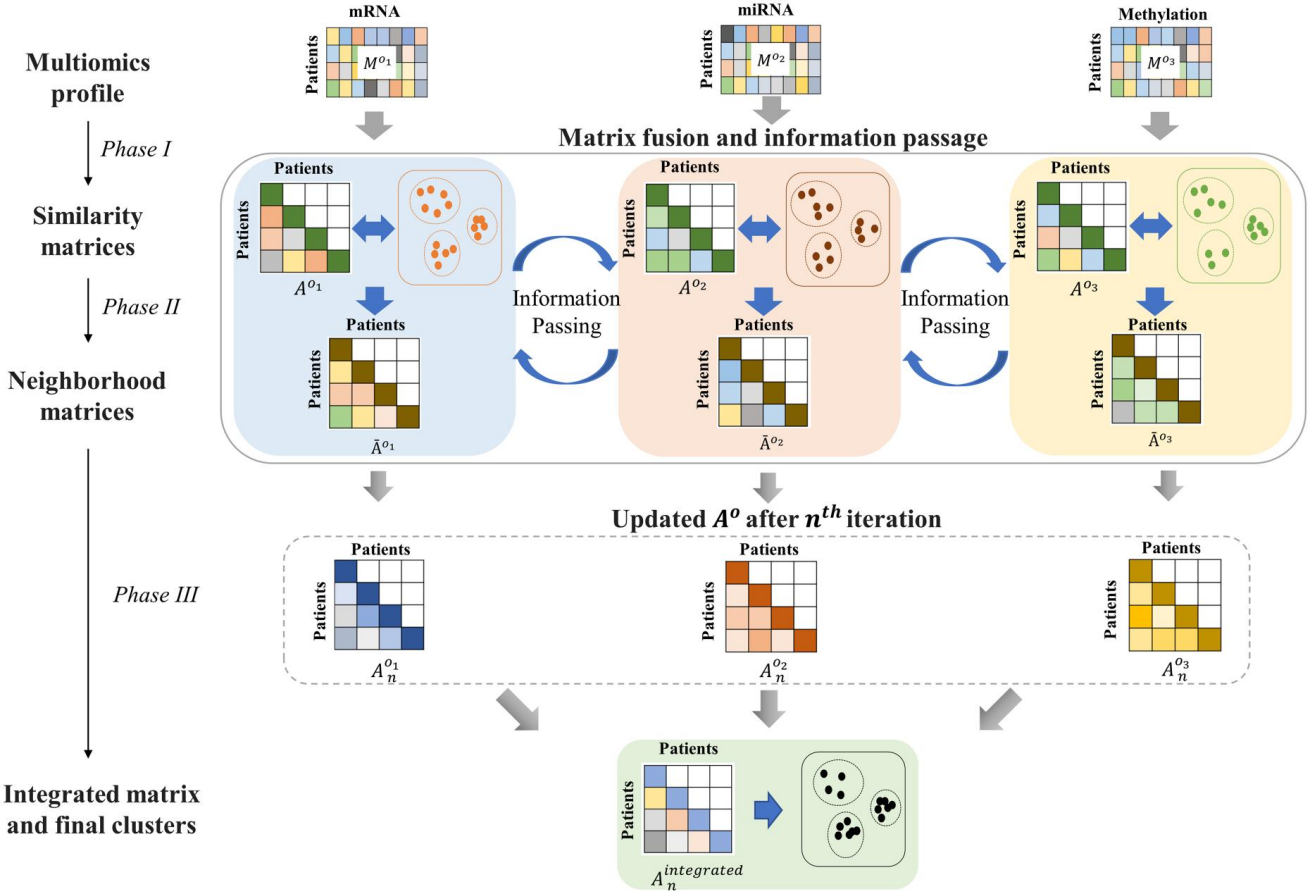
Schematic illustration of *i*CluF workflow for calculating pairwise similarity matrices (Phase I), deriving neighborhood matrices and message passing across data types iteratively (Phase II), and integrating updated similarity matrices to identify final clusters (Phase III).

*i*CluF focuses on the integration of multiomics data and the identification of clusters without using any prior knowledge, such as known associations or labels. This methodology is divided into three phases: (i) calculation of the patient–patient similarity matrix for each omic profile; (ii) calculation of neighborhood profile similarities for each omic profile, passing information between each omic matrix, and updating original similarity matrices iteratively; and (iii) integration of updated similarity matrices and derivation of final clusters. In Phase 3, all updated similarity matrices are averaged to generate a final similarity matrix, which is further used to produce the final clusters, as schematically illustrated in Fig. 1. The detailed steps of the working pipeline are described below:

*Phase 1*: For each omics data type, we defined the data matrix $M_{n,f}^{o}$, where n is the total number of patients, f is the total number of features, and o ∈ {miRNA, mRNA, methylation data types} in this study. In matrix$\;M_{\;}^{o_{\;}}$, in each row, $p_{i}^{o}$ represents the feature profile of patient i in omic type o. In cases of more than three omic datasets, the extended matrices can be defined accordingly. We used Euclidean distance to measure the profile similarity $A_{ij}^{o}\;$between patients i and j, as defined below:


(1) $$A_{ij}^{o}\;=e^{{-\alpha \left(\left|\left|p_{i}^{o}-p_{j}^{o}\right|\right|\right)}^{2}.}$$

The formulation of (1) is similar to the Gaussian Interaction Profile kernel, which has been widely used in previous studies that focused on similarity-based scoring configurations (Lan *et al.* 2017, Jiang *et al.* 2019, Nguyen *et al.* 2021, Shakyawar *et al.* 2022). In this study, it measures the similarity between two patients using the Euclidean distance between them. The hyperparameter, α, in (1) regularizes the bandwidth of the kernel and thus, regulates the decay of the similarity scores curve indicating patients with higher distances between them are less similar than the ones that are closer. The value for the hyperparameter α is set empirically. We computed the similarity scores with varying α values in the range of 1.5–7.5, on a set of Euclidean distances between two patients (Supplementary Fig. S1). Based on the observation that the scores were more differentiated for lower values of α, we used α=1.5 to perform further experiments with the current cancer datasets.

*Phase 2*: During the second phase, we calculated the neighborhood profile similarity, ${Ā}_{ij}^{o}$, between patients i and j. For each omic type, we calculated ${Ā}_{ij}^{o}$ for each pair of patients, resulting in an omic-specific neighborhood profile matrix, which incorporates information from the original patient–patient similarity matrix and *K*-means clusters, which were produced using $A_{ij}^{o}$. The neighborhood similarities intermediately improve patient(s) classification by incorporating cluster properties, such as inter-cluster distances and the distance of patient(s) from the centroid of any neighboring cluster. ${Ā}_{ij}^{o}$ can be defined as follows:


(2) $${Ā}_{ij}^{o}=\frac{2\times {\left[A_{ij}^{o}\right]}^{2}}{D\left(c_{i},c_{j}\right)\left\{µ+\frac{1}{n}\sum_{q=1}^{n}\mathrm{Dist}\left(v_{i},q\right)+\frac{1}{m}\sum_{q=1}^{m}\mathrm{Dist}\left(v_{j},q\right)\right\}}.$$

Regarding, patient i ∈ cluster $c_{i}$, and patient j ∈ cluster $c_{j}$, the clusters were derived using spectral clustering at given *K* (number of clusters).

$v_{i}$ and $v_{j}$ are centroids of clusters $c_{i}$ and $c_{j}$, respectively.

m and n are the total number of patients in clusters $c_{i}$ and $c_{j}$, respectively.

$\mathrm{Dist}\left(v_{i},q\right)$ is the distance between patient q (in cluster $c_{i}$) from centroid $v_{i}$. Similarly, the $\mathrm{Dist}\left(v_{j},q\right)$ is defined for patient q with respect to cluster $c_{j}$.

µ is a parameter that was introduced to avoid singularity in (2). Theoretically, there is a possibility that all the neighboring points may coincide on their corresponding centroids (i.e. $\mathrm{Dist}\left(v_{i},q\right)=\;\mathrm{Dist}\left(v_{j},q\right)=\;0$), if relatively higher number of iterations are chosen. The positive value of µ in the denominator of (2) avoids any such singularity in computation of ${Ā}_{ij}^{o}$. Therefore, we empirically set µ = 1 for all the experiments performed in the study.

Using the Euclidean distance between the centroids, $v_{i}$ and $v_{j}$, of clusters $c_{i}$ and $c_{j}$, respectively, the inter-cluster separation measure $D\left(c_{i},c_{j}\right)$ is defined below:


(3) $$D(c_{i},c_{j})=e^{\frac{{\beta \left(\left|\left|v_{i}-v_{j}\right|\right|\right)}^{2}}{\eta_{c_{i},c_{j}+1}}.}$$

The parameter β in (3), regulates exponential raise of inter-cluster separation measure $D\left(c_{i},c_{j}\right)$, which further helps in converging neighborhood profile similarity matrices ${Ā}_{ij}^{o}$, and subsequently derives more compact and homogenous clusters, as depicted in (2). Based on our experiments, we recommend lower range of β values (0.1–1), which can better control exponential increment in the calculation of $D\left(c_{i},c_{j}\right)$.

$\eta_{c_{i},c_{j}}\;$ works as a normalizing factor to reduce scaling bias for the inter-cluster distance, as described in Wang *et al.* (2014), and is calculated as follows:


$$\eta_{c_{i},c_{j}}\;=\frac{\frac{1}{n}\sum_{q=1}^{n}\mathrm{Dist}\left(v_{i},q\right)+\frac{1}{m}\sum_{q=1}^{m}\mathrm{Dist}\left(v_{j},q\right)+\left|\left|v_{i}-v_{j}\right|\right|}{3}.$$

*Phase 3*: During Phase 3, we updated the omic-specific similarity matrices, $A^{o}$, over multiple iterations (Fig. 1). Following the strategy in Wang *et al.* (2014), $A^{o}$ at the *n*th iteration can be defined as follows:


(4) $$A_{n}^{o_{1}}=\;{Ā}_{n-1}^{o_{1}}\times \;A_{n-1}^{o_{2}}\times \;{\left[{Ā}_{n-1}^{o_{1}}\right]}^{T}+\;{Ā}_{n-1}^{o_{1}}\times \;A_{n-1}^{o_{3}}\times \;{\left[{Ā}_{n-1}^{o_{1}}\right]}^{T}$$

where ${\left[{Ā}_{n-1}^{o_{1}}\right]}^{T}$is the transpose of ${Ā}_{n-1}^{o_{1}}$.

Similarly, $A_{n}^{o_{2}}$ and $A_{n}^{o_{3}}$, for omic data types $o_{2}$ and $o_{3},\;$respectively, were calculated. In this case, we denoted the mRNA, miRNA, and methylation data using notations $o_{1}$, $o_{2}$, and $o_{3}$, respectively. Based on our experiments on convergence, measured through gradual increase in Silhouette scores of the *i*CluF-predicted clusters using 10 randomly chosen cancer types with wide range of sample sizes (Supplementary Fig. S2), we observed that the number of iterations around seven has provided more observable and homogeneous clusters. An average of the three matrices $A_{n}^{o_{1\;}}$, $A_{n}^{o_{2\;}}$, and, $A_{n}^{o_{3\;}}$ was calculated to generate integrated matrix $A_{n}^{\mathrm{integrated}}$, as defined below. We finally adopted *K*-means to derive the final clusters.


(5) $$A_{n}^{\mathrm{integrated}}\;=\frac{A_{n}^{o_{1\;}}+\;A_{n}^{o_{2\;}}+\;A_{n}^{o_{3\;}}}{3}$$

.

### 2.3 Evaluation metrics for clustering assessment

We used multiple metrics to evaluate the model’s prediction performance and compare it with previous approaches. We calculated the Silhouette score of the predicted clusters to assess the homogeneity of the subtypes. Additionally, we used the adjusted rand index (ARI) and Fowlkes–Mallows (FM) score to assess the performance of each model to predict the BRCA subtypes. Furthermore, we compared *i*CluF with some recent methods and the most used methods, such as SNF, iClusterPlus, PINSPlus, and *K*-means, as these also work for multiomics integration when predicting subtypes with significant survival differences. We identified the clusters using each of these methods and their default parameters (*K* = 2, 3, 4, and 5), and we performed Cox regression to assess survival differences between the resulting subtypes. The *P*-values from the log-rank test were compared across 30 cancer types. We also measured and compared each method’s runtime for predicting the ACC subtypes at *K* = 2, 3, 4, and 5 to understand the computational complexity and implementation requirements. All the programs were run on a server with 8 GPUs and a combined 512 GB of RAM.

### 2.4 Evaluation of model performance using different combinations of omic data types

*i*CluF was implemented on multi-level omics information to understand patients’ level correlation and identify clusters with different molecular and clinical features. To evaluate model’s performance on different combinations of omic types, we prepared the following subsets of BRCA data with 849 samples.

Subset 1—omic data type: miRNA and methylation

Subset 2—omic data type: miRNA and mRNA

Subset 3—omic data type: mRNA and methylation.

We ran *i*CluF to identify clusters (at *K* = 5) using all the above data subsets, individually. Next, we computed the significance of survival differences of the predicted clusters in each case and compared the *P*-value. We also compared *i*CluF’s performance with the other methods when the above data subsets are used as input. For this, we measured and compared the significance of survival difference of the clusters predicted by other methods including SNF, iClusterPlus, PINSPlus, and *K*-means.

### 2.5 Features importance analysis and assessment

We adopted the Gini importance (GI) scoring scheme from the state-of-the-art method Random Forest (RF) (Nembrini *et al.* 2018) to assess the contributions of different omic data types when predicting *i*CluF subtypes across all cancers. In this case, we included *i*CluF-predicted subtypes at a *K* (=3). For each omic data type, the feature matrices with expression values and *i*CluF-predicted subtypes were used as an input for the RF model, which outputs the importance score of each feature in each omic dataset. We selected the top 25% of features in each omic type and we added the sum of their importance scores, followed by the min./max. normalization, to perform inter-omic comparisons of contributions from each omic type.

## 3 Results and discussion

### 3.1 Testing the performance of *i*CluF using datasets from 30 cancer types

**Figure 2.**
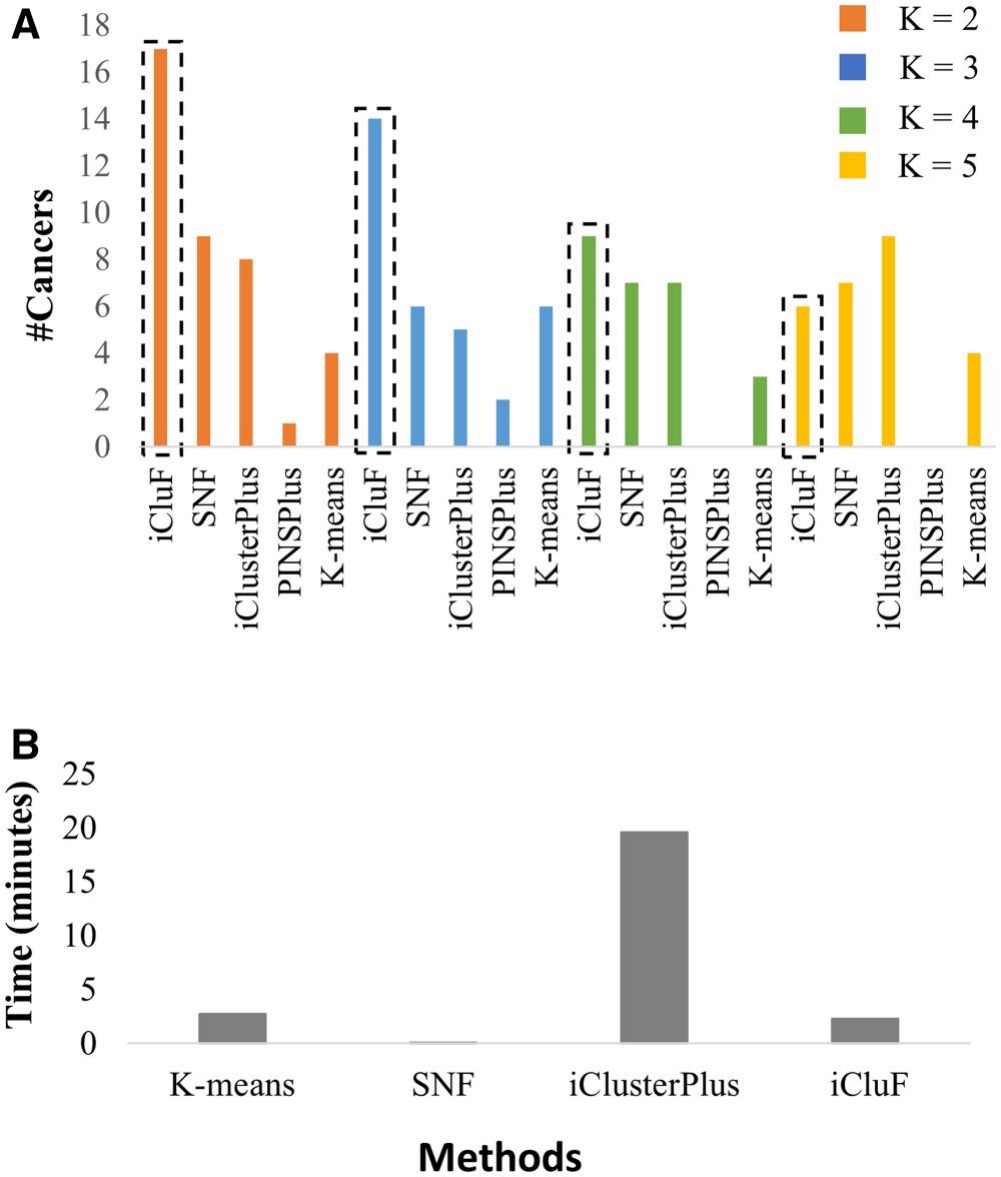
(A) Comparison of various methods for predicting cancer subtypes with significant survival differences. (B) Comparison of running times of different methods for predicting ACC subtypes. The running time of different methods was averaged over *K* = 2, 3, 4, and 5, for predicting ACC subtypes. PINSPlus is not included in this comparison as it does not predict any subtypes at *K* = 4 and 5.

We used multiomics datasets from 30 different cancers obtained from TCGA to develop and test *i*CluF because multiomics datasets obtained from the same patient are readily available at TCGA, and it demonstrates the robustness of *i*CluF against a large number of disease datasets. The cancer types used, and the number of samples and omic features used in each cancer type, are shown in Supplementary Table S1. We used the miRNA, mRNA, and methylation features of each cancer type to generate similarity matrices and derive integrated clusters using *i*CluF. For each cancer type, we identified clusters using *i*CluF and four other comparable methods (SNF, iClusterPlus, PINSPlus, and *K*-means) at *K* = 2, 3, 4, and 5. A comparison was made based on how many cancer types (out of 30) have their clusters (subtypes) predicted with significant survival differences in each *K*-means group across the five methods. This metric has been used previously to evaluate similar method (Speicher and Pfeifer 2015). As described in the methods section, the *P*-value from the Cox log-rank test shows the significance of the differences between the survival profiles of the predicted clusters. Our results showed that *i*CluF outperformed other methods at *K* = 2, *K* = 3, and *K* = 4. The predicting clusters showed significant differences between survival rates in the 17, 14, and 9 cancer types, respectively (Fig. 2A). However, at *K* = 5, iClusterPlus performed better than all other methods by identifying significant clusters in nine cancer datasets, whereas *i*CluF performed moderately well (in six cancers) in this scenario.

The superior performance of *i*CluF for all but the *K* = 5 cluster is attributable to the following novel aspects of our approach: (i) feature selection strategy and (ii) iterative message passing and integration of similarity matrices. By comparison, other methods use either concatenation-based, generalized linear model-based, or network fusion-based integration strategies. In particular, the SNF method that is most similar to our approach uses very strict edge weight criteria for refining similarity networks, which may lose important node (i.e. patients) connections in the patient–patient similarity network. *i*CluF overcame this limitation by not losing any connections while iterating and integrating similarity matrices during the intermediate steps (Phase II). The passage of information across different omics-derived similarity matrices is the most crucial step in the integration process, contributing to the clustering performance of *i*CluF. The patient–patient similarity matrices are generated using each omic data type individually. In multiple iterations, the similarity score between two patients either decreases or increases when information is passed across different omic-based similarity matrices (Phase II). This simulation technically provides a strong basis for downstream clustering process, which further reflects the impact of most relevant omic features. Moreover, other methods also suffer from a lack of effective feature selection criteria prior to cluster identification, leading to the possible inclusion of noise in the pairwise similarity calculation process. From the literature-based discussions, feature selection helps reduce the dimensionality of matrices by retaining only data points that are relevant to the predictions; however, in many cases, this strategy prior to cluster identification is also interpreted as one of the major limitations, as it might unintentionally exclude important data points (Li *et al.* 2018, Pudjihartono *et al.* 2022, Shakyawar *et al.* 2022). In this study, we used stringent criteria for feature selection, which retain only the features that are most relevant in pan-cancer, as discussed in other studies (Hoadley *et al.* 2018, Yang *et al.* 2021). Therefore, our feature selection strategy is also important in calculating patient–patient similarity matrices using individual omic datasets (Phase I), which further reflects their impression in the integration process (Phase II).

Furthermore, we observed that *i*CluF was the only predictor of clusters with significant survival differences in certain cancer types, which shows the robustness of the method. For example, in six cancer types (HNSC, LUAD, LUSC, COAD, PAAD, and UCS), *i*CluF was the only method that identified two clusters (*K* = 2) with significant survival differences. Similarly, at *K* = 3, only *i*CluF-predicted significant clusters in UCEC, STAD, SARC, ESCA, PAAD, and UCS datasets (Supplementary Table S2). LUCS, KIRP, UVM, and ACC were the cancer types for which subtypes were predicted using only *i*CluF at *K* = 4. In the same way, at *K* = 5, though it underperformed compared with iClusterPlus, *i*CluF uniquely identified cancer types (BLCA, STAD, LUSC, and PCPG) with significant survival differences. Counting all *K*s, across an average of five cancer datasets, only *i*CluF was able to predict the clusters with significant differences (*P*-value <.05) in their survival profiles; this is higher than other methods considered in this study (Supplementary Table S2). Interestingly, out of all the methods, only *i*CluF and *K*-means identified five clusters with significant survival differences, with *P*-values of .006 and .02, respectively, in the BRCA dataset. Supplementary Figure S3B shows that Kaplan–Meier survival plot of the *i*CluF-predicted five BRCA subtypes.

Additionally, our analyses of algorithm running times showed that the average runtime of *i*CluF (over *K* = 2, 3, 4, and 5 on ACC dataset) is 2.5 min, which is almost equivalent to that of *K*-means, and slightly higher than SNF (Fig. 2B). We omitted PINSPlus in this comparison as no subtype predictions were made for ACC at *K* = 2, 4, and 5. The running time of *i*CluF is much lower than that of iClusterPlus, showing the favorable computational complexity of the method, especially when multi-level data are integrated iteratively.

### 3.2 Comparison of *i*CluF and *K*-means clustering using survival analysis

**Figure 3.**
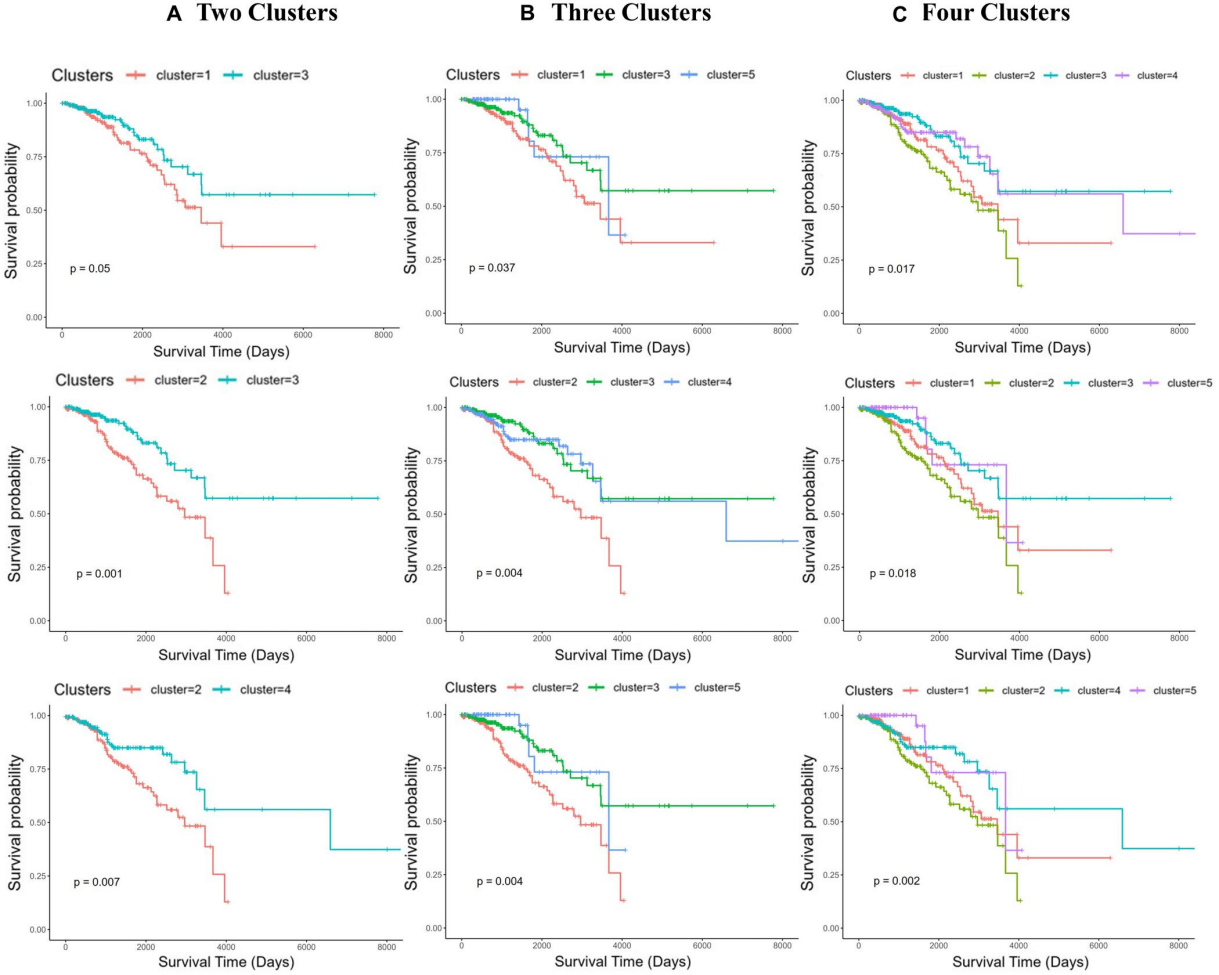
Kaplan–Meier survival plots of randomly chosen combinations of (A) two, (B) three, and (C) four clusters predicted by *i*CluF using the BRCA dataset with 849 samples including 139 Basal, 52 HER2-enriched, 463 Luminal A, 160 Luminal B, and 35 Normal subtypes.

As the log-rank test provides a single *P*-value for the combined survival differences between the clusters, we were interested to test the significance of the survival differences between different combinations of clusters within the five-clusters predicted with *K* = 5 using BRCA data. *i*CluF and *K*-means are the only methods that predicted significant survival differences with *K* = 5, so we ran this experiment to compare these two methods. We generated Kaplan–Meier estimates for all possible combinations of the two, three, and four cluster groups (within the five clusters of *i*CluF or *K*-means at *K* = 5), resulting in 10, 10, and 5 estimates, respectively (Supplementary Table S3). In all the cluster groups, *i*CluF outperformed *K*-means by showing significant survival differences between more cluster groups that were generated in random combinations (Supplementary Fig. S4). Kaplan–Meier survival plots of top three performing clusters in each category (two clusters, three clusters, and four clusters) are shown in Fig. 3. This demonstrates the robustness of the *i*CluF methodology as it discriminates between the clusters; this occurs because the algorithm uniquely captures information from each omic modality, and it fuses them iteratively. As hypothesized, our mechanism can utilize multi-level omic information more holistically than *K*-means, which mainly relies upon a concatenation-based integration strategy.

### 3.3 Predictions of BRCA’s intrinsic and receptor-based subtypes

We further analyzed and compared predicted subtypes against the intrinsic subtypes of BRCA because among all of the cancer datasets, BRCA has the most well-characterized clinical subtypes, as follows: Luminal A, Luminal B, human epidermal growth factor receptor 2 (HER2)-enriched, Basal, and Normal. The proper classification of patients with BRCA into these subtypes are crucial for developing effective and more personalized treatment strategies. Therefore, we compared five *i*CluF-predicted clusters (at *K* = 5) against the clinical subtypes of BRCA using the ARI and FM score (Table 1). In this scenario, we achieved an ARI of 0.71 and a FM score of 0.81, which showed a good concordance between *i*CluF-predicted and clinical subtypes. The calculated Silhouette score, in this case, was 0.89, which reflects a fairly good compactness and homogeneity of the predicted clusters (Supplementary Fig. S5A). The predicted clusters also showed significant differences between their survival profiles, as demonstrated by the Kaplan–Meier survival plot of five *i*CluF-predicted BRCA subtypes (Supplementary Fig. S3B). Regarding BRCA, normal subtypes generally show similarities with the normal breast epithelium. This may be because of the low amount of tumor cells collected in the biopsy. Therefore, these subtypes are clearly not classified as distinctive and independent tumor types (Larsen *et al.* 2013). This might be the reason why several computational studies primarily emphasized predicting only four BRCA subtypes (Basal, HER2, Luminal A, and Luminal B) instead of five (Jaber *et al.* 2020, Phan *et al.* 2021, Zhang *et al.* 2023). We also ran *i*CluF with *K* = 4 after removing the normal subtype from training, and we only predicted four clinical subtypes. In this scenario, *i*CluF performed slightly better, achieving ARI and FMI scores of 0.72, and 0.83, respectively (Table 1). The good homogeneity of the clusters, in this case, is shown with the calculated Silhouette score of 0.86 (Table 1 and Supplementary Fig. S5B). These analyses are further indicative of *i*CluF’s powerful integration strategy for merging BRCA’s multiomic datasets and predicting subtypes at different *K*s.

**Table 1.** Performance measurements of *i*CluF for predicting BRCA’s subtypes using different evaluation scores.^a^

Dataset		*i*CluF predictions		
	*K*	ARI	FM score	Silhouette score
Dataset 1	5	0.71	0.81	0.89
Dataset 2	4	0.72	0.83	0.86

a Dataset 1 includes Basal (139 samples), HER2-enriched (52 samples), Luminal A (463 samples), Luminal B (160 samples), and Normal (35 samples); Dataset 2 is a subset of Dataset 1 without normal samples. *K*, number of clusters; ARI, adjusted rand index; FM, Fowlkes–Mallows.

We also compared *i*CluF’s performance when different combinations of omic data types from BRCA data were used to train the model, as described in the methodology. We recorded highest significance (*P*-value =.006) in the survival difference among the predicted clusters when all three omic data types (i.e. miRNA, mRNA, and methylation) (Supplementary Fig. S3B) are used, as compared to when Subset 1 (miRNA and methylation), Subset 2 (miRNA and mRNA), and Subset 3 (mRNA and methylation) are used as input with calculated *P*-values as .13, .23, and .92, respectively (Supplementary Fig. S3A). The survival difference of all five predicted clusters in case of Subsets 1, 2, and 3 is shown in Kaplan–Meier plots in Supplementary Fig. S3C–E, respectively. In neither case, we observed clusters with significant difference in their survival profile except when combination of all three omic data types were used, showing the importance of incorporating multi-level omic information for clustering patients. Further, we compared *i*CluF’s performance with methods SNF, iClusterPlus, and *K*-means, to predict clusters with survival differences when Subsets 1, 2, and 3 were used in training (Supplementary Fig. S6). Results showed that *i*CluF was the best predictor even though insignificant (*P*-value =.13), when Subset 1 was used, while *K*-means scored lowest in this case. With Subset 2, SNF and *K*-means performed almost equally but better than *i*CluF and iClusterPlus. Only *K*-means were able to identify significant clusters with *P*-value =.03 and .02 on data subset 2 (miRNA and mRNA) and complete dataset (miRNA, mRNA, and methylation), respectively. Among all comparisons, it can be clearly seen that *i*CluF achieved the highest significance (*P*-value =.006) when all three omic data types (miRNA, mRNA, and methylation) were used as input features to the model.

Traditionally, BRCA subtypes were defined based on the expression status of the estrogen receptors, progesterone receptors, and HER2. With current cancer therapies, discordance between receptor-based subtypes and PAM50 gene panel-based subtypes has been recorded in around 20%–50% of patients (Paquet and Hallett 2015, Kim *et al.* 2019, Dix-Peek *et al.* 2023), which could result in the wrong administration of treatment for BRCA patients. Therefore, it is extremely important to perform BRCA-correct classification that satisfies both subtyping strategies. We tested *i*CluF to predict the receptor-based characterization of all intrinsic subtypes of BRCA, separately. We divided the patients into each intrinsic subtype (gene panel-based) by considering receptor status, and we created positive/negative instances for each subtype (further descriptions concerning the classification and final counts for each instance are provided in Supplementary Table S4). *i*CluF achieved ARI and FM scores of 0.78 and 0.89, respectively, which was the best-case scenario for predicting positive/negative instances of HER2 subtypes (Table 2). For Basal, Luminal A, and Luminal B, we achieved ARI scores of 0.68, 0.72, and 0.75, respectively. All of the predicted clusters demonstrated good homogeneity, as shown by the Silhouette score (Table 2), further indicating *i*CluF’s capability to perform binary classifications, as shown in Fig. 2A. We believe that this level of in-depth characterization, concerning the heterogeneity between different subtypes, may provide additional support for the stratification of BRCA patients in clinical settings.

**Table 2.** Evaluation of *i*CluF’s predictions using intrinsic and receptor-based subtypes.

BRCA subtypes (samples)	Positive/negative instances	*i*CluF predictions		
		ARI^a^	FM^a^ score	Silhouette score
Basal (139)	103/36	0.68	0.88	0.93
Luminal A (463)	349/114	0.72	0.90	0.96
Luminal B (160)	126/34	0.75	0.92	0.94
Her2 (52)	27/25	0.78	0.89	0.95

a ARI, adjusted rand index; FM, Fowlkes–Mallows.

### 3.4 Contributions of different omic features for predicting cancer subtypes

**Figure 4.**
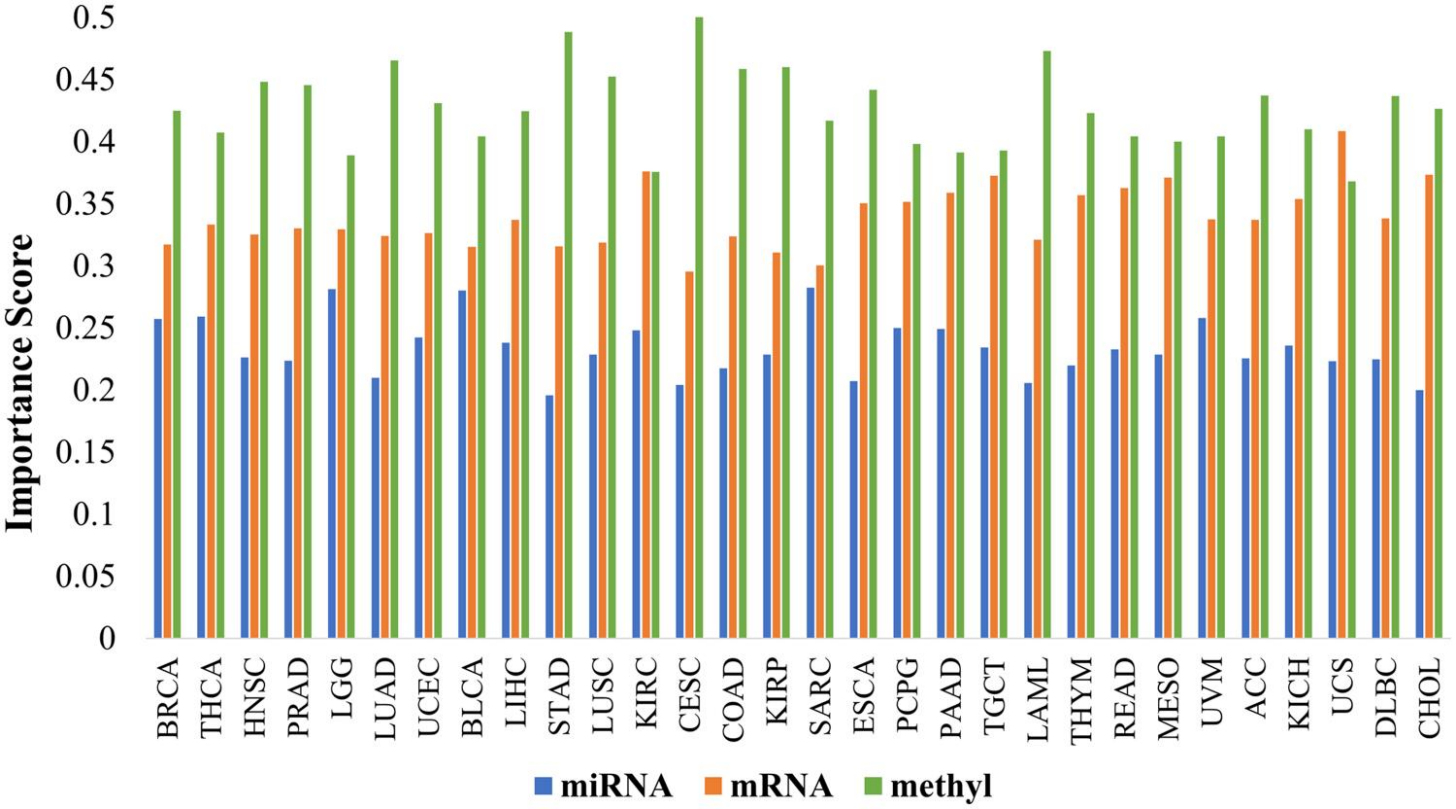
Comparison of contributions of omics features: miRNA, mRNA, and methylation for predicting subtypes using TCGA’s cancer dataset. The GI scores of omic features were calculated by using *i*CluF-predicted clusters at *K* (number of clusters) = 3 and original feature matrices. methyl: methylation.

Feature importance estimation is essential for understanding the contribution proportion of each data type for the model’s predictions. In the present context, our objective included the relative assessment of the contributions of miRNA, mRNA, and methyl features when predicting *i*CluF subtypes in different cancers. As described in the methods section, GI scoring schemes estimate the importance of variables (i.e. features, as in our study) when maintaining the homogeneity of the nodes in trees (of RF). Our calculated GI distributions of omic features (calculated from *i*CluF predictions at *K* = 3) showed that miRNAs, with an average score of 0.23 were the least contributing features across all cancer types except SARC, where miRNA and mRNA features contributed almost equally for predicting the subtypes (Fig. 4). In BRCA, miRNA, mRNA, and methylation features achieved GI scores as 0.26, 0.32, and 0.42, respectively, showing their respective decisive roles in identifying subtypes. Across all cancer types, the highest GI scores of miRNA, mRNA, and methylation features were 0.28 (in SARC), 0.41 (in UCS), and 0.50 (in CESC), respectively (Fig. 4). The mRNA and methylation features achieved an average GI score of 0.34 and 0.43, respectively, showing comparatively higher contributions when predicting subtypes in most of the cancer types. Only for UCS, MESO, TGCT, and KIRC did mRNAs score higher or almost equally to the methylation features; conversely, in other cancer types, the methylation dataset dominated the cluster predictions (Fig. 4).

Furthermore, we assessed similarities between cancer types based on commonly identified top contributing features (miRNA, mRNA, and methylation) in each cancer. The result showed higher similarities (but not more than 50%) between cancer types when methylation features were compared to mRNA features (Supplementary Fig. S7). Fewer similarities based upon miRNA features were observed between different cancer types, which is likely due to the low number of features. We observed that the cancer types BRCA, LUAD, HNSC, CESC, and STAD show comparatively higher similarities between each other in the context of methylation features. Similarly, LUSC, KIRC, and PAAD showed similar ranges of similarity scores. When the top mRNA features were compared, cancer types, such as COAD, STAD, ESCA, and READ showed high discordance in terms of their similarities at the gene level (Supplementary Fig. S7). Similarly, KIRO, DLBC, and THYM are highly dissimilar in terms of their mRNA profiles. These comparative analyses are helpful to understand the commonalities and differences between various cancer types at different omic levels.

Though *i*CluF can identify cancer subtypes with distinct clinical features, the method uses geometric distance norms when calculating patient–patient similarity; this means that it might not be able to handle data noise for this operation. The prior selection of the number of subtypes to run *i*CluF is also a limitation, as compared with other non-parametric methods. Similarly, the use of *K*-means may ignore dimensional differences between multiple omic datasets.

## 4 Conclusions

The identification of cancer subtypes and their molecular characterization is essential for developing more personalized treatment strategies. Our method, *i*CluF, implements an iterative integration strategy for extracting and combining commonalities across multiomic datasets to derive clusters with distinct molecular features. We tested our method on TCGA’s 30 cancer datasets to predict subtypes that differ in terms of their survival profiles. Our methodology is easy to implement for other diseases with multi-cohort patient information because our integration strategy is more robust in terms of capturing variability with regard to similarities when multi-level information is involved. In this study, only three omic data types were used for model prediction, but users can seamlessly include more omic data types. This approach also captures and integrates hidden biological information in the data by including the correlations between the omic datatypes via message passing while building the neighborhood matrices; this may help improve the accuracy of subtype predictions. Furthermore, our feature importance analysis helped us understand the relative decisive roles of different omic modalities for predicting cancer subtypes.

## Supplementary Material

vbae015_Supplementary_Data

## Data Availability

*i*CluF was developed using Python (version 3.7.6). Users can download the program via the GitHub link https://github.com/GudaLab/iCluF_core and they can follow the ReadMe document to run the code. All the relevant input data and output files are in the data folder. All Supplementary Files are provided in the Supplementary Folder.
