# Supplementary material for: iCluF: an unsupervised iterative cluster-fusion method for patient stratification using multiomics data
Source: Bioinform Adv. 2024 Jan 30;4(1):vbae015. doi: 10.1093/bioadv/vbae015 (PMC11063539; doi:10.1093/bioadv/vbae015)
Supplement: vbae015_Supplementary_Data [file vbae015_supplementary_data.zip › iCluF_Supplementary_final.docx]

***i*CluF: An unsupervised iterative cluster-fusion method for patient stratification using multiomics data**

Sushil K Shakyawar^1^, Balasrinivasa R Sajja^2^, Jai Chand Patel^1^, Chittibabu Guda^1,3 *^

^1^Department of Genetics, Cell Biology and Anatomy, University of Nebraska Medical Center, Omaha, NE 68198

^2^Department of Radiology, University of Nebraska Medical Center, Omaha, NE 68198

^3^Center for Biomedical Informatics Research and Innovation, University of Nebraska Medical Center, Omaha, NE 68198

***Correspondence to:** Chittibabu Guda [babu.guda@unmc.edu](file:///\\Users\Babu.Guda\Dropbox\BABU\Manuscripts\PREPARATION\CURRENT\COVID-Sushil\Final\babu.guda@unmc.edu)

Professor & Vice Chair for Bioinformatics Research & Training,

Department of Genetics, Cell Biology and Anatomy,

University of Nebraska Medical Center, Omaha, NE 68198-5805, United States

E-mail: [babu.guda@unmc.edu](mailto:babu.guda@unmc.edu), Phone: (402) 559-5954

# **Omics data preprocessing**

***miRNA data***: We downloaded miRNA read counts for all the samples from TCGA using TCGAbiolinks (Colaprico, et al., 2016). The miRNAs with a value 0 in more than 20% of samples were removed. The normalized counts were generated using DESeq2 package.

***mRNA data***: Similar to miRNA, the read counts of mRNAs were downloaded using TCGABiolinks and filtered if any features had a value of 0 in more than 20% of samples, followed by conversion of read counts to normalized values using DESeq2.

***Methylation data***: The beta values corresponding to each CpGs were downloaded from TCGA. We removed CpGs with 0 or NA values in more than 20% of the samples. Next, we used imputeKNN (K = 10) to impute missing beta values in the matrix.

The final counts of samples and omic features in each cancer type are mentioned in **Table S1**.


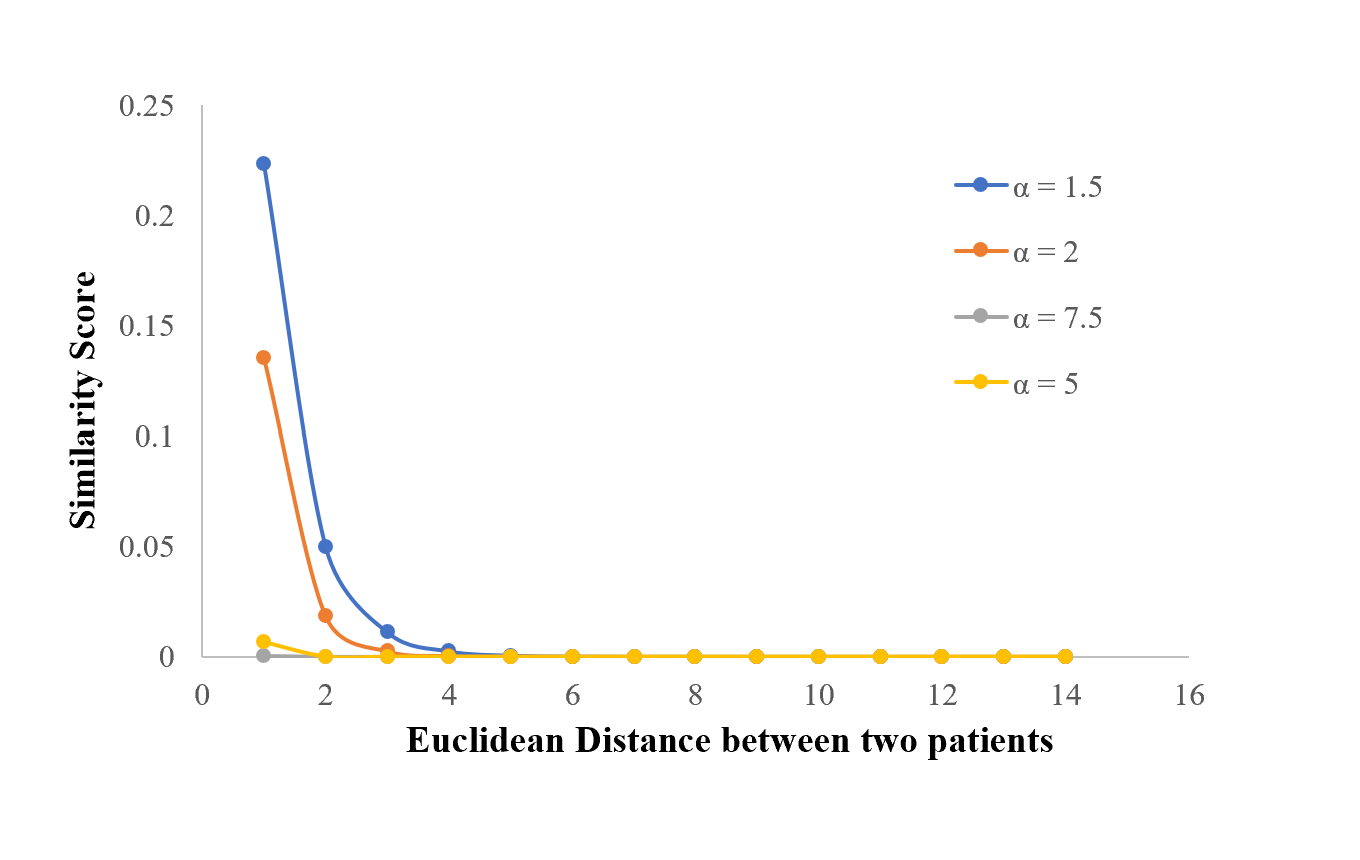


**Figure S1**: Effect of variation in hyperparameter, $\alpha,$ on calculation of profile similarity $A_{ij}^{o}$ between patients $i$ and $j$, on a given set of Euclidean distances.


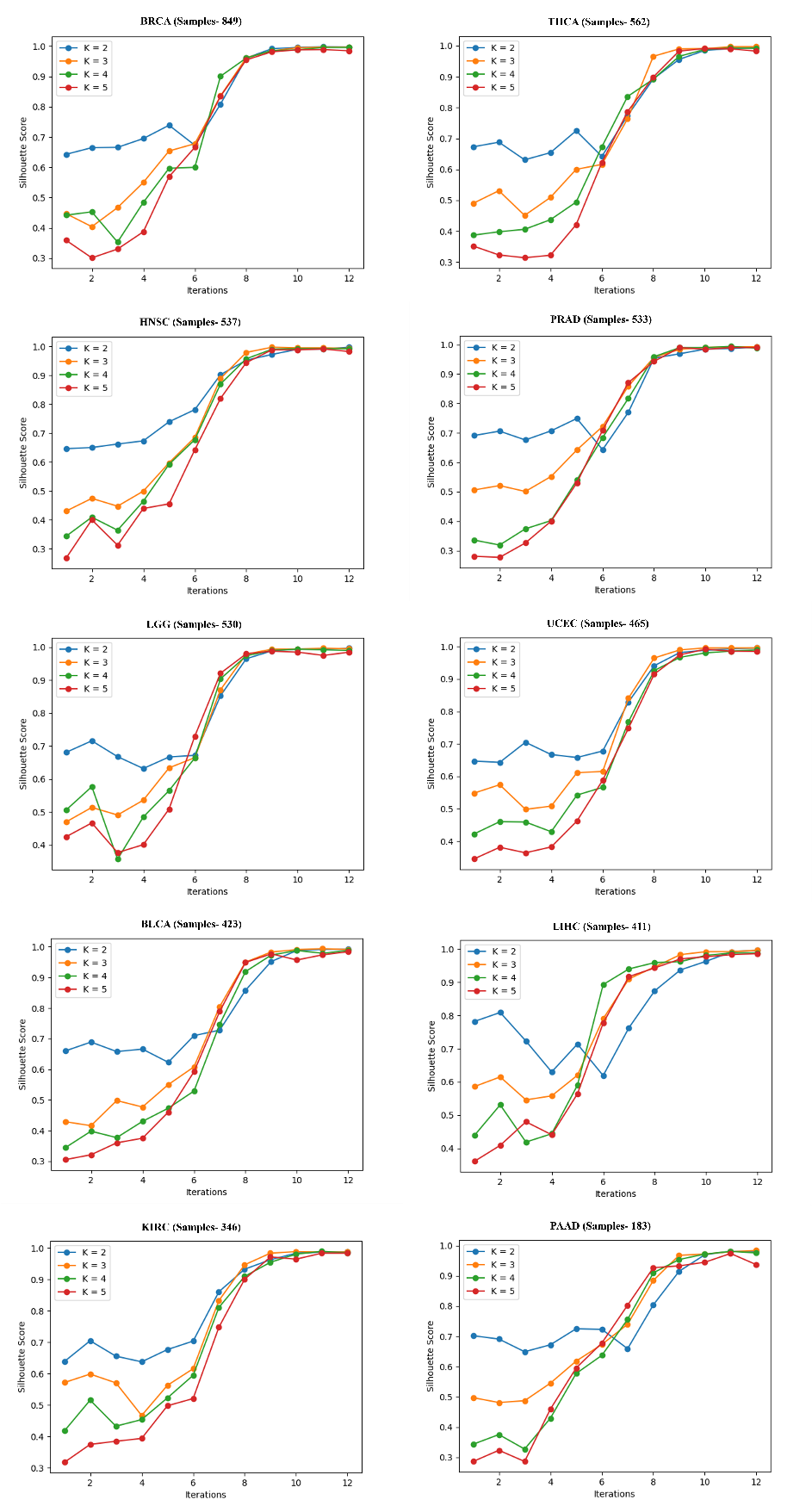


**Figure S2:** Convergence of similarity matrices observed through gradual increase in Silhoutte scores, representing homogeneity of the *i*CluF-predicted clusters in different cancer types. We ran iCluF using 10 randomly chosen cancer types with a diverse range of samples (183 to 849), and predicted clusters at K = 2, 3, 4, and 5. The Silhouette score was measured at each K for 12 iterations.


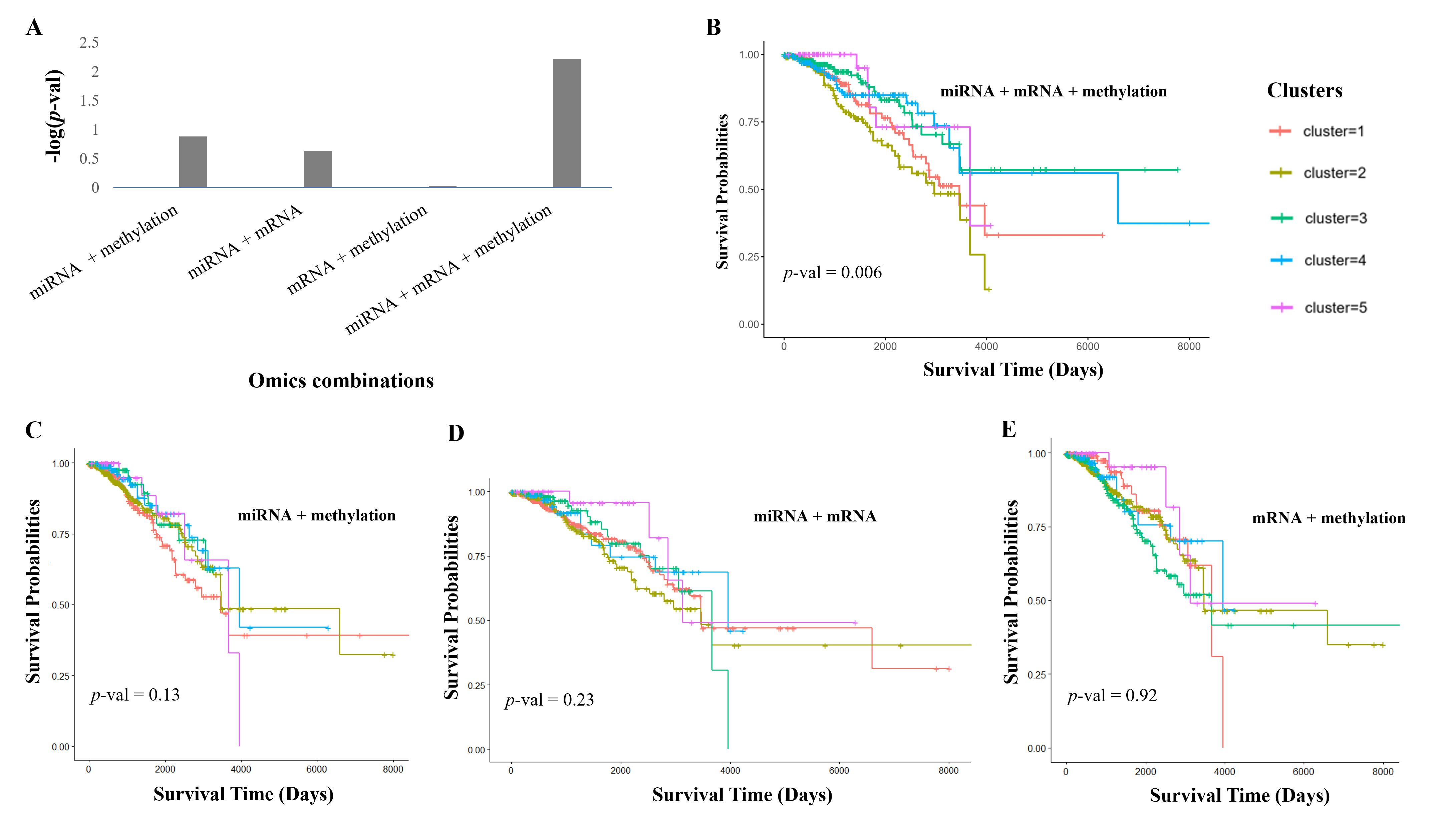


**Figure S3**: A) Comparison of the significance of survival differences of the predicted BRCA’s five subtypes when different omic data types were used as input to iCluF at K = 5. Kaplan-Meier survival plots of iCluF predicted five subtypes of BRCA when we used B) miRNA, mRNA, and methylation; C) miRNA and methylation; D) miRNA and mRNA, and E) mRNA and methylation as input. The number of samples in each case was the same at 849.


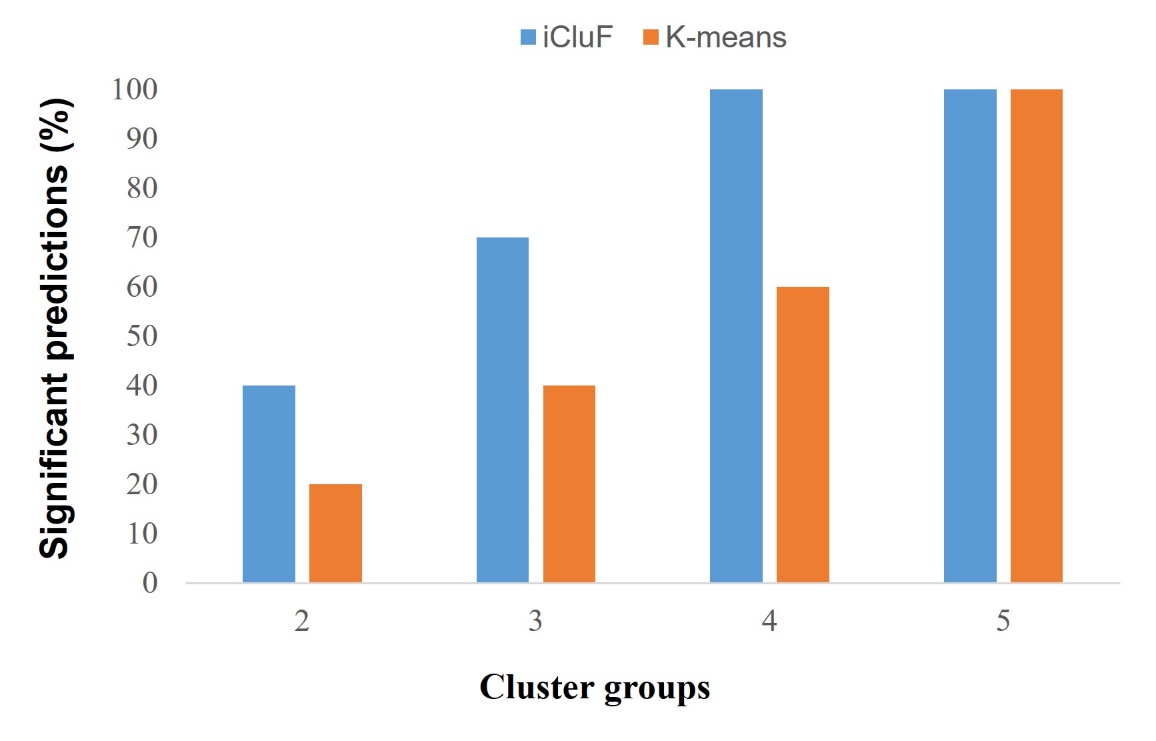


**Figure S4:** Performance comparison of *i*CluF and K-means on random clusters group combinations. The clusters were generated using the BRCA dataset at K = 5.


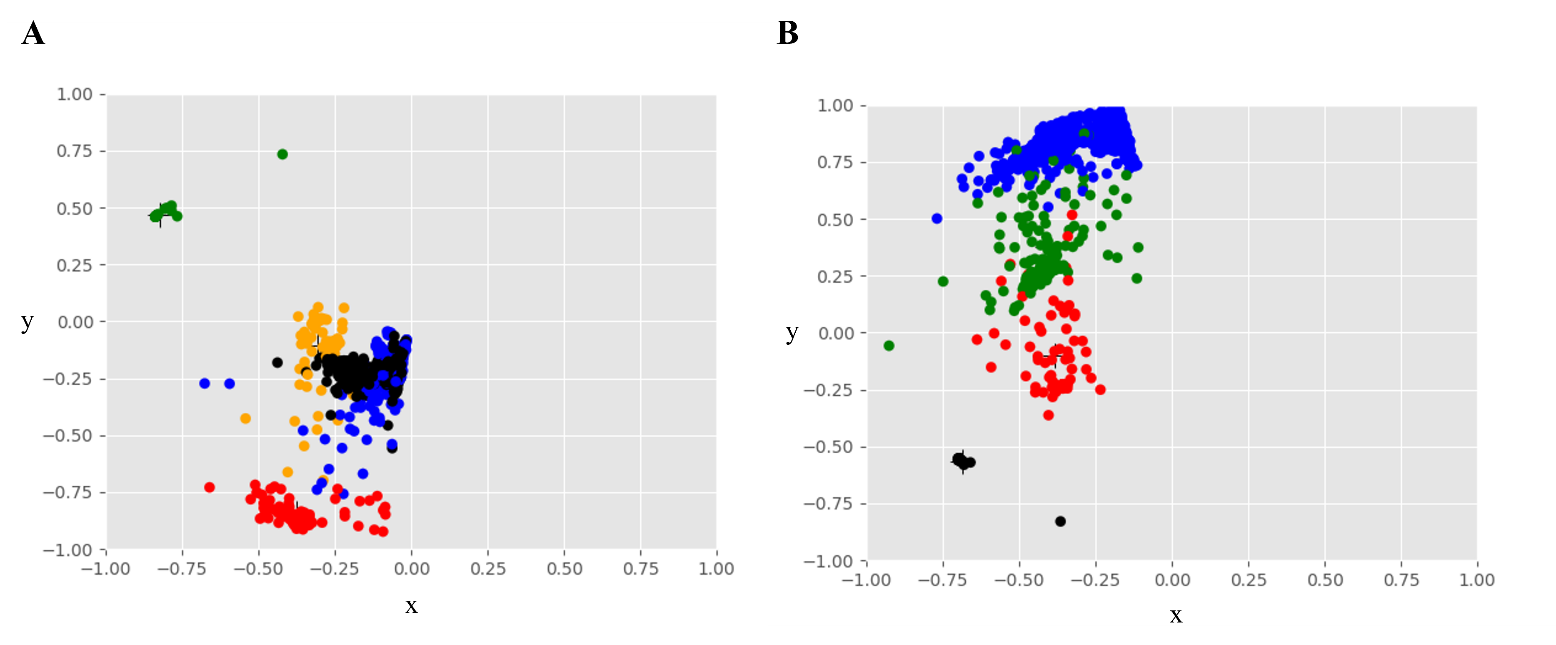


**Figure S5:** Clusters plot of iCluF predicted subtypes of BRCA when **A)** Dataset 1 (849 samples) is used and iCluF is run at K = 5, and **B)** Dataset 2 is used and iCluF is run at K = 4. Dataset 1 includes Basal (139 samples), Her2 (52 samples), Luminal A (463 samples), Luminal B (160 samples), Normal (35 samples); Dataset 2 is a subset of Dataset 1 without normal samples.

***
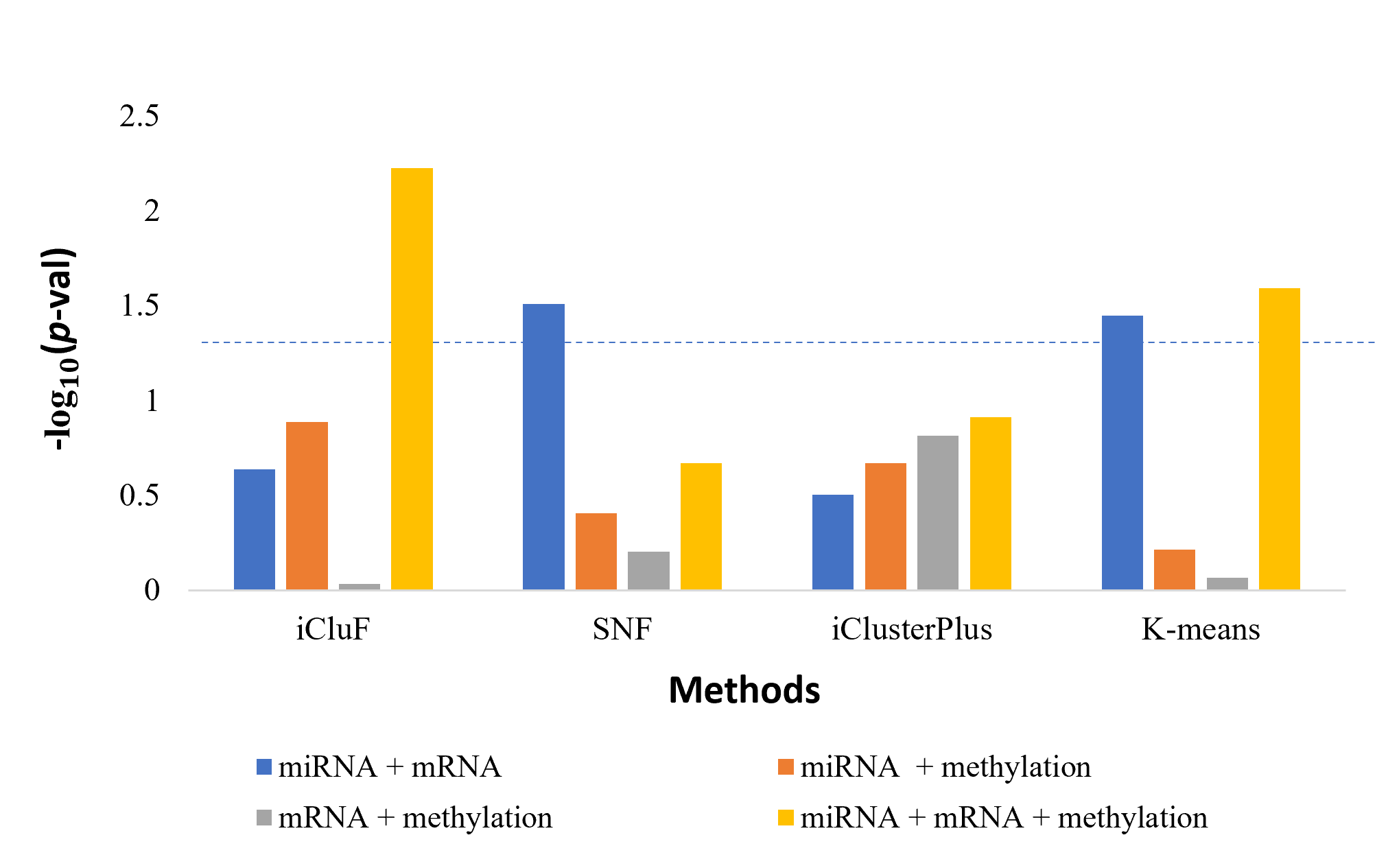
***

**Figure S6:** Performance comparison of *i*CluF with other methods for predicting BRCA’s subtype with significant survival difference, when different combinations of omic data types are used as input to train the different models. We ran *i*CluF at K = 5. The total number of BRCA samples used in each case was 849. Same dataset and subsets were used to train all other methods such as SNF, iClusterPlus, and K-means with their default settings. PINSPlus did not show any results therefore not considered in the figure. The dotted line in light blue represents the significance level at *p*-val <0.05. Subset 1 (miRNA and methylation), subset 2 (miRNA and mRNA), subset 3 (mRNA and methylation), and complete dataset (miRNA, mRNA, and methylation).


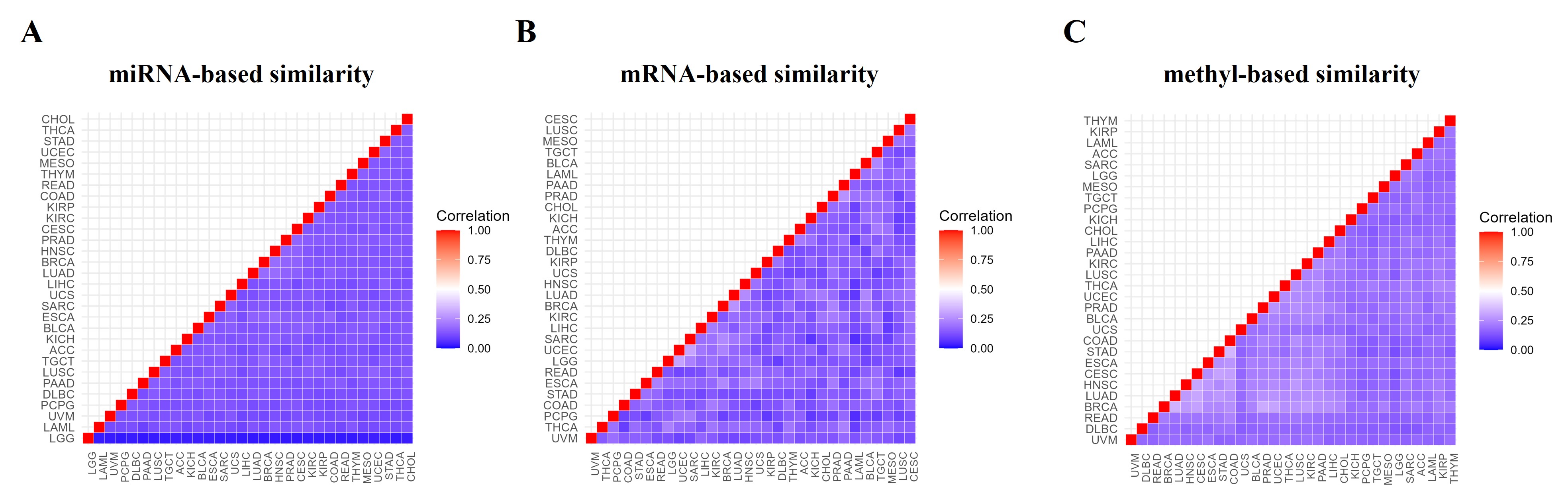


**Figure S7:** Similarities between different cancer types based on miRNA (A), mRNA (B), and methyl (C) features. Top 25% features were selected based on importance scores and common features were compared across cancer types to calculate similarity scores.

# **References**

Colaprico, A.*, et al.* TCGAbiolinks: an R/Bioconductor package for integrative analysis of TCGA data. *Nucleic Acids Res* 2016;44(8):e71.
